# Rapid detection of *Burkholderia cepacia* complex carrying the *16S rRNA* gene in clinical specimens by recombinase-aided amplification

**DOI:** 10.3389/fcimb.2022.984140

**Published:** 2022-09-05

**Authors:** Hanyu Fu, Lin Gan, Ziyan Tian, Juqiang Han, Bing Du, Guanhua Xue, Yanling Feng, Hanqing Zhao, Jinghua Cui, Chao Yan, Junxia Feng, Zheng Fan, Tongtong Fu, Ziying Xu, Rui Zhang, Xiaohu Cui, Shuheng Du, Yao Zhou, Qun Zhang, Ling Cao, Jing Yuan

**Affiliations:** ^1^ Department of Bacteriology, Capital Institute of Pediatrics, Beijing, China; ^2^ Department of Pulmonology, The Affiliated Children’s Hospital, Capital Institute of Pediatrics, Beijing, China; ^3^ Institute of Hepatology, Chinese People Liberation Army General Hospital, Beijing, China

**Keywords:** *Burkholderia cepacia* complex, recombinase-aided amplification, rapid detection, 16S rRNA, infection

## Abstract

The *Burkholderia cepacia* complex (BCC) is a group of opportunistic pathogens, including *Burkholderia cepacia, Burkholderia multivorans, Burkholderia vietnamiensis* and *Burkholderia ambifaria*, which can cause severe respiratory tract infections and lead to high mortality rates among humans. The early diagnosis and effective treatment of BCC infection are therefore crucial. In this study, a novel and rapid recombinase-aided amplification (RAA) assay targeting the *16S rRNA* gene was developed for BCC detection. The protocol for this RAA assay could be completed in 10 min at 39°C, with a sensitivity of 10 copies per reaction and no cross-reactivity with other pathogens. To characterize the effectiveness of the RAA assay, we further collected 269 clinical samples from patients with bacterial pneumonia. The sensitivity and specificity of the RAA assay were 100% and 98.5%, respectively. Seven BCC-infected patients were detected using the RAA assay, and three BCC strains were isolated from the 269 clinical samples. Our data showed that the prevalence of BCC infection was 2.60%, which is higher than the 1.40% reported in previous studies, suggesting that high sensitivity is vital to BCC detection. We also screened a patient with *B. vietnamiensis* infection using the RAA assay in clinic, allowing for appropriate treatment to be initiated rapidly. Together, these data indicate that the RAA assay targeting the *16S rRNA* gene can be applied for the early and rapid detection of BCC pathogens in patients with an uncharacterized infection who are immunocompromised or have underlying diseases, thereby providing guidance for effective treatment.

## Introduction

The *Burkholderia cepacia* complex (BCC) is a group of over 20 phenotypically similar but genetically different Gram-negative, non-fermenting, bacteria that includes *Burkholderia cepacia*, *Burkholderia multivorans*, *Burkholderia vietnamiensis* and *Burkholderia ambifaria* (Depoorter et al., 2016; Sfeir, 2018; Tavares et al., 2020). Members of the BCC are opportunistic pathogens that can cause severe respiratory infections in immunocompromised patients (such as those with congenital immunodeficiency, HIV, and cancer patients receiving chemotherapy) or patients with underlying diseases such as cystic fibrosis (CF) and chronic granulomatous (Kalish et al., 2006; Greenberg et al., 2009; Tavares et al., 2020). According to a previous study in Nepal, the infection rate of BCC was 1.4% in patients who received more than 48 hours of mechanical ventilation (Baidya et al., 2021). The 2017 CF Foundation Annual Report on Patient Registrations showed that the occurrence of BCC was 2.4% in CF patients (Blanchard and Waters, 2019). Although the BCC only accounts for a small proportion of lung infections in CF patients, BCC infection accelerates lung function deterioration, which leads to a poor prognosis and high mortality (Whiteford et al., 1995; Stephenson et al., 2015). This high mortality is generally caused by the progression of “cepacia syndrome”, which is clinically characterized by necrotizing pneumonia, sepsis, hyperthermia, and even severe progressive respiratory failure (Lord et al., 2020; Lauman and Dennis, 2021). In view of the significant morbidity and mortality associated with BCC infection, rapid and accurate detection is vital to initiate timely and effective treatment. Currently, BCC identification mainly involves isolation on selective media and DNA-based detection methods such as PCR and qPCR (De Volder et al., 2021). However, novel methods that offer high sensitivity and specificity, while being quick and easy to perform, are much needed (Ahn et al., 2020).

Recombinase-aided amplification (RAA) is a rapid, specific, sensitive and reliable isothermal gene amplification technology (Wang et al., 2021). At present, RAA has been widely used in the detection of various pathogens such as SARS-CoV-2, adenovirus, hepatitis B virus, *Escherichia coli*, *Klebsiella pneumoniae*, *Mycoplasma pneumoniae*, *Salmonella* and *Vibrio parahaemolyticus* (Zhang et al., 2017; Shen et al., 2019; Wang et al., 2019; Xue et al., 2020; Mu et al., 2021; Zhang et al., 2021; Zheng et al., 2021; Feng et al., 2022). RAA achieves DNA amplification by employing recombinase UvsX, DNA polymerase and single-stranded DNA binding protein at 35°C–42°C, replacing the traditional thermal cycling process (Fan et al., 2020). With the addition of 6-carboxyfluorescein (FAM)-labeled probes and biotin-labeled primers, double-labeled amplification products can be obtained, making the assay sensitive and specific (He et al., 2021).

In this study, pairs of primers and probes targeting the *16S rRNA* gene, which is highly conserved and specific for BCC species, were designed, and a rapid and intuitive RAA assay with high sensitivity and specificity for BCC detection was established. To assess the applicability of the RAA assay in clinic, 269 clinical samples were detected, and conventional PCR was also performed for comparison.

## Material and methods

### Ethical approval

This study was performed in compliance with the Helsinki Declaration (Ethical Principles for Medical Research Involving Human Subjects) and was approved by the research board of the Ethics Committee of the Capital Institute of Pediatrics, Beijing, China (SHERLLM2022004). All specimens used in this study are part of routine patient management without any additional collection, and all patient data were anonymously reported.

### Bacterial strains

The RAA assay was evaluated with 12 clinically-common pathogens, including *Haemophilus influenzae*, *Mycobacterium tuberculosis*, *Staphylococcus aureus*, *Pseudomonas aeruginosa*, *K. pneumoniae*, *E. coli*, *M. pneumoniae*, *B. cepacia*, *B. multivorans*, *B. vietnamiensis*, *B. ambifaria* and *B. gladioli*. The details of these strains are shown in **Table S1**.

### DNA extraction and BCC isolation of clinical samples

A total of 269 bronchoalveolar lavage fluid, throat swab or sputum samples from patients with bacterial pneumonia were randomly collected. Total DNA was extracted from the 269 collected samples using the QIAamp DNA Mini Kit (Qiagen, Hilden, Germany) and was stored at −80°C. BCC standard strains and the clinical samples were plated onto *B. cepacia* selective agar (BCSA) for 2 days at 35°C, followed by 3 days of incubation at room temperature (Martina et al., 2020).

### Recombinant plasmid construction

The PCR amplification product of the *16S rRNA* gene (*BCC*, GenBank accession number: LC496395) was cloned into the vector pGM-T (Tiangen Biotech, Beijing, China) according to the manufacturer’s instructions. The primers used for amplification are shown in **Table 1**. The concentration of plasmid was detected using a NanoDrop spectrophotometer. Then, the standard recombinant plasmids were prepared at 10-fold dilutions ranging from 10^6^ copies/µL to 10^0^ copies/µL for sensitivity analysis. The plasmid concentration and copy number were determined using the formula: DNA copy number (copy number/µL) = [6.02×10^23^ × plasmid concentration (ng/µL) ×10^−9^]/[DNA length (in nucleotides) × 660]. The length of the DNA (3226 bp) was the sum of the plasmid fragment length (211 bp) and the vector length (3015 bp).

**Table 1:** The primer sequences used in the study.

Primer	Sequence (5’ to 3’)	Function
*16S*-F1	GCAGGCTAGAGTATGGCA	conventional PCR
*16S*-R1	GTTACTAAGGAAATGAATCCC	conventional PCR
*16S*-1-F	TAAGACMGATGTGAAATCCCCGGGCTCAACC	RAA assay
*16S*-1-R	GCTGCCTTCGCCATCGGTATTCCTCCACATCT	RAA assay
*16S*-1-P	CTAGAGTATGGCAGAGGGGGGTAGAATTCCACG [FAM-dT][THF][BHQ-dT]AGCAGTGAAATGCGT	RAA assay

### RAA primer design

The sequence of the *16S rRNA* gene was used for RAA primer design. According to the principles of RAA primer and probe design (primer length between 30 to 35 bp, probe length between 46 to 52 bp), four sets of primers and probes were manually designed in highly-conserved regions. Primer and probe specificities were analyzed using NCBI Primer-BLAST, and the formation of primer dimers and secondary structures (hairpins) was predicted using the Website OligoEvaluator (http://www.oligoevaluator.com/LoginServlet). The primers and probes were synthesized and purified using high-performance liquid chromatography (Sangon Biotech, Shanghai, China).

### RAA assay

The RAA assay was performed in a 50 μL reaction volume using a commercial RAA kit (Qitian, Jiangsu, China). The reaction mixture included 2 μL extracted DNA template, 25 μL reaction buffer, 15.7 μL DNase-free water, 2.1 μL primer F/R, 0.6 μL of probe and 2.5 μL of magnesium acetate. The reaction mixture was added to the tube containing the RAA enzyme mixture in lyophilized form. The test tubes were placed in a B6100 shaking mixer (QT-RAA-B6100, Qitian), incubated for 4 min, mixed briefly, centrifuged, and finally transferred to a fluorescence detector (QT-RAA-1620, Qitian), and measured at 39°C for 10 min.

### Sensitivity and specificity of the RAA assay

The sensitivity of the RAA assay was assessed using a series of diluted recombinant plasmids ranging from 10^6^ to 10^0^ copies/μL. The specificity was evaluated by detecting four strains of different BCC subspecies, one strain of *B. gladioli* (which does not belong to the BCC) and seven clinically-common pathogens, as presented in **Table S1**. The positive control was *16S rRNA*-positive plasmid, and the negative control was nucleic acid-free water.

### PCR assay

PCR primers were designed according to primer design principles (**Table 1**). The conventional PCR assay was performed in a 25 μL reaction system containing 12.5 μL 2 × Taq PCR MasterMix (Tiangen Biotech), 1.0 μL of each primer and 2 μL of extracted DNA template. The reaction program was set as 95°C for 10 min, followed by 35 cycles of 30 s at 95°C, 30 s at 55°C and 1 min at 72°C, and a final extension step for 10 min at 72°C. PCR products were visualized on a 1.5% agarose gel and stained with GeneGreen. Images were acquired with a Gel Doc EQ imaging system (Bio-Rad, Hercules, USA), and the amplified product was sequenced (Sangon Biotech).

### Evaluating the RAA assay on clinical samples

Established RAA assay was evaluated using 269 clinical samples from patients with bacterial pneumoniae. The RAA assay results were compared to conventional PCR assay. The formulas for evaluating RAA assay were as follows: consistency between RAA and conventional PCR assays = (true positives + true negatives)/(true positives + true negatives + false positives + false negatives) × 100%, sensitivity of RAA assay = true positives/(true positives + false negatives) × 100%, specificity of RAA assay = true negatives/(false positives + true negatives) × 100%, and the conventional PCR method was used as gold standard for identification (Gautam et al., 2017). A case was also used for assessing the clinical application of RAA assay for detecting BCC infection in this study. The pus sample extracted from the patient’s neck abscess and sputum were used for BCC isolation, RAA and metagenomic sequencing (Vison Medicals, Guangzhou, China).

### Genome sequencing and analysis

BCC isolates were subjected to DNA extraction using the Wizard Genome DNA Purification Kit (Promega, Madison, USA) and sequenced on the Illumina HiSeq PE150 platform (Novogene, Beijing, China). The genome sequences were assembled and annotated by software SOAP denovo and Prokka. Multilocus sequence typing (MLST) identification was performed using the Institute Pasteur database (https://bigsdb.pasteur.fr/klebsiella/klebsiella.html) by analyzing the sequences of seven housekeeping genes (*atpD*, *gltB*, *gyrB*, *recA*, *lepA*, *phaC* and *trpB*) (Diancourt et al., 2005). The core genome single-nucleotide polymorphisms (SNPs) of BCC strains were detected using the software Snippy and Gubbins, as previously described (Gan et al., 2022). The extracted SNPs were used for phylogenetic tree construction by software MEGA. Strains MSMB384WGS and FL_2_3_10_S3_D0 were used as reference strains for *B. cepacia* phylogenetic tree and *B. vietnamiensis* phylogenetic tree, respectively (Croucher et al., 2015). Strain ATCC10248 (*B. gladioli*) was included as an outgroup. All strains used for constructing the phylogenetic tree are listed in **Table S2**.

## Results

### Primers and probes designed for the RAA assay

**Figure 1:**
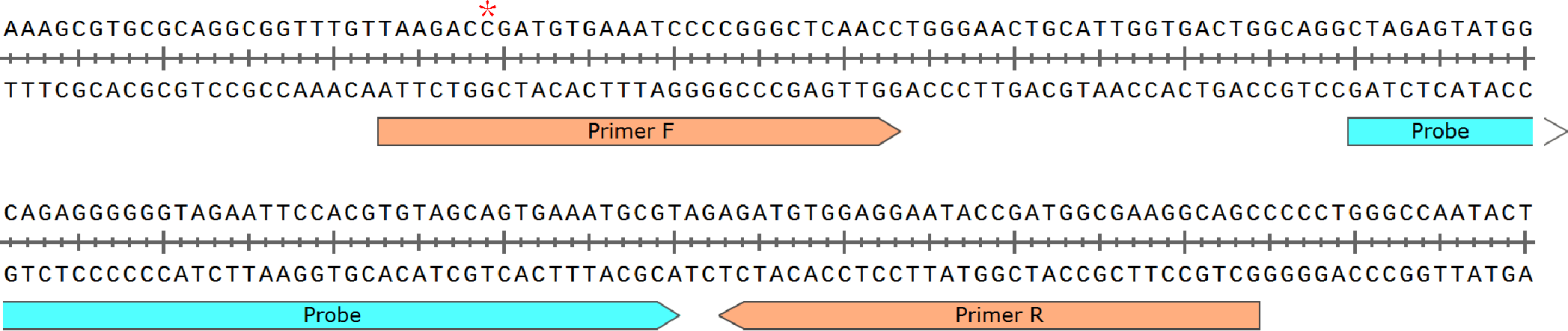
Primer and probe sequences used in RAA detection. * denotes the site with multiple subtypes between BCC species, *Burkholderia cepacia* is C, *Burkholderia multivorans* is A or C, *Burkholderia vietnamiensis* is C, and *Burkholderia ambifaria* is C.

Four sets of RAA primers and probes named *16S*-1 (**Figure 1** and **Table 1**), *16S*-2, *16S*-3 and *16S*-4 (**Table S3**) were manually designed against the conserved region of the *16S rRNA* gene of BCC. No cross-reactivity with other species was predicted by the BLAST tool. The amplification efficiencies of the four sets of primers and probes were compared, and the recombinant *16S rRNA* plasmid was used as the positive template and nucleic acid-free water was used as the negative template. All reactions were performed in triplicate. Under the same reaction condition and system, *16S*-1 exhibited the highest amplification efficiency compared to other sets (**Supplementary Figure 1**). Therefore, *16S*-1 was chosen as the primers and probe for the subsequent analysis.

### Sensitivity of the RAA assay

**Figure 2:**
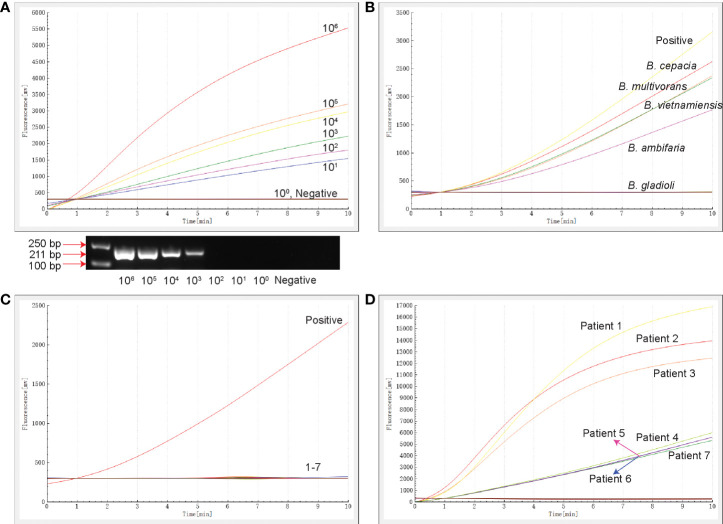
The sensitivity, specificity and clinical testing of the RAA assay. **(A)** The sensitivity of RAA and conventional PCR assays. The results of RAA are shown by an amplification curve, and the PCR amplification products are shown following separation on a 1.5% agarose gel. **(B)** The specificity of the RAA assay for members of the *Burkholderia* family. **(C)** The specificity of RAA for clinically-common pathogens. 1: *Haemophilus influenzae* ATCC10211, 2: *M. tuberculosis* ATCC25618, 3: *Staphylococcus aureus* ATCC29213, 4: *Pseudomonas aeruginosa* ATCC27853, 5: *K. pneumoniae* ATCC BAA-2146, 6: *E. coli* ATCC25922, 7: *Mycoplasma pneumoniae* M129 ATCC 29342. **(D)** Seven samples tested positive by the RAA assay. The positive control was *16S rRNA*-positive plasmid, and the negative control was nucleic acid-free water.

The template concentration of the recombinant plasmid determined by the NanoDrop spectrophotometer was 153.6 ng/µL, and the converted copy number was 4.34×10^10^ copies/µL. The 10-fold serially diluted recombinant plasmids ranged in concentration from 10^6^ to 10^0^ copies/µL. The results of sensitivity analysis are shown in **Figure 2A**. The RAA assay detected the *16S rRNA* gene within 10 min at 39°C at a sensitivity of 10 copies/µL. The minimum concentration of conventional PCR was 10^3^ copies/µL.

### Specificity of the RAA assay

The specificity of the RAA assay was confirmed by the detection of four strains of different BCC subspecies and eight clinically-common pathogens (**Table S1**). Only *B. cepacia*, *B. multivorans*, *B. vietnamiensis* and *B. ambifaria* produced fluorescence signals (**Figure 2B**), while the other strains tested, including *B. gladioli* that does not belong to the BCC, were negative (**Figures 2B**, **C**). No cross-reactivity with strains of other species being detected in this RAA assay targeted *16S rRNA*.

### Evaluation of the RAA assay using clinical samples

A total of 269 specimens collected from patients with bacterial pneumonia were simultaneously detected by RAA and conventional PCR assays (**Table 2**). Seven samples (2.6%) tested positive and 262 samples tested negative for the BCC by the RAA assay (**Figure 2D**), whereas three cases (1.1%) tested positive and 266 cases tested negative in conventional PCR assay. Among them, four samples were positive by RAA and negative by the conventional PCR assay. The percentage consistency between RAA and PCR assays was 98.5%. Compared with the gold standard PCR method, the RAA assay had a sensitivity of 100% and a specificity of 98.5%.

**Table 2:** Testing of clinical samples.

Results	PCR positive	PCR negative	Total
RAA positive	3	4	7
RAA negative	0	262	262
Total	3	266	269

To investigate the value of the RAA assay in terms of clinical application, we also analyzed a patient with *B. vietnamiensis* infection in detail. The 7-year-old girl was diagnosed with chronic granulomatous disease. To determine the cause of infection, cervical lymph node puncture sample was collected for metagenomic sequencing, and the results indicated *B. vietnamiensis* infection. Meanwhile, BCC infection was further confirmed by the RAA assay targeting the *16S rRNA* gene, and a BCC isolate was successfully isolated from a pus sample, confirming the reliability of RAA assay for clinical diagnosis.

**Figure 3:**
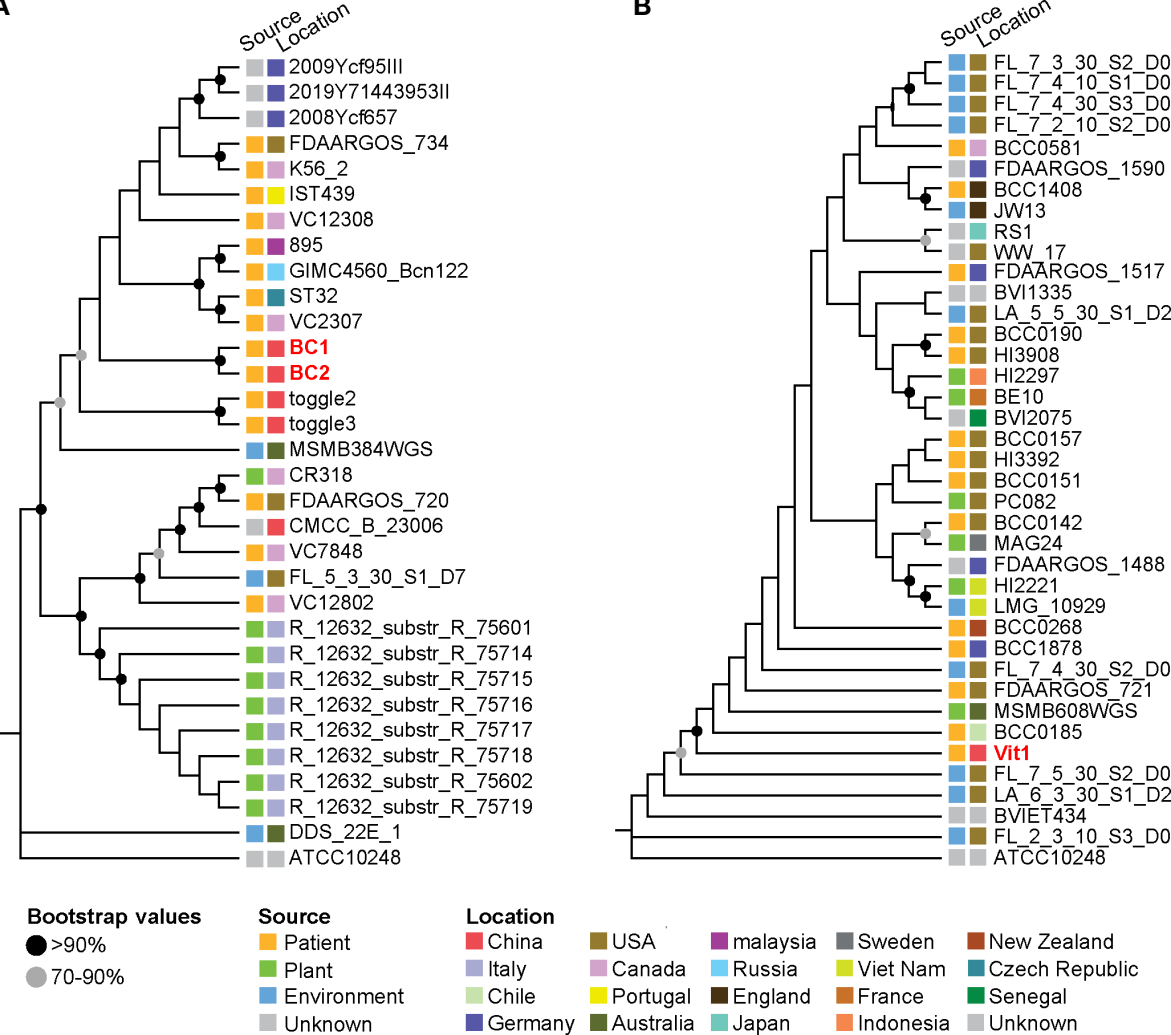
Phylogenetic trees of *Burkholderia* strains based on core single-nucleotide polymorphisms. **(A)** Phylogenetic tree of *B. cepacia* strains. **(B)** Phylogenetic tree of *B. vietnamiensis* strains. The squares beside the strains denote the source and location of the strains.

The prevalence of BCC infection among patients with pneumonia was 2.60%, and three strains were isolated from these positive samples. Isolates BC1 (ST608, Genome accession number SAMN29388125) and BC2 (ST608, Genome accession number SAMN29388126) belonged to *B. cepacia*, and isolate Vit1 (Genome accession number SAMN29388124) belonged to *B. vietnamiensis* (*atpD* type 27, *gltB* type 231, *gyrB* type 16, *recA* type 22, *lepA* type 12, *phaC* 5 type 6 and *trpB* type 268, which was a new sequence type). In the phylogenetic tree of *B. cepacia*, isolates BC1 and BC2 clustered with strains collected from patients in China (toggle2 and toggle3), suggesting that these four strains from China were closely related phylogenetically and potentially shared epidemiological characteristics (**Figure 3A**). In the *B. vietnamiensis* phylogenetic tree, isolate Vit1 was separate from the Chinese strains, indicating that it shared no phylogenetic or epidemiological characteristics (**Figure 3B**).

## Discussion

BCC pathogens are an important cause of secondary severe pulmonary infection in patients with a congenital immunodeficiency or existing underlying disease, and earlier identification of pathogens means more rapid intervention and therefore better clinical outcomes. However, BCC pathogens typically require 48 to 72 hours of incubation for colonies to appear on selective media (Brown and Govan, 2007). Currently, BCC identification mainly depends on molecular methods (Drevinek et al., 2008), but the sensitivity of PCR assays varies widely. Moreover, BCC diagnosis also faces challenges posed by non-BCC *Burkholderia* species, such as *B. gladioli* (Brown and Govan, 2007), and a detection method is urgently needed to distinguish these organisms. In recent years, RAA has been successfully applied to the detection of many pathogens and SNPs (Qin et al., 2021). Because of its fast speed, low cost and high sensitivity, RAA is potentially suitable for clinical application.

Multiple copies of *rRNA* genes are present throughout the bacterial genome. Therefore, *16S rRNA* gene-based PCR assays generally have higher sensitivity than PCR assays targeting single-copy genes such as *recA* (Brown and Govan, 2007). In this study, the gene *16S rRNA* of BCC was selected through detailed analysis of conserved regions in multiple sequence alignments. The *16S rRNA* gene shows no cross-homology and meets the requirements for designing BCC-specific RAA primers. The specificity and sensitivity of the RAA primers were verified by BLAST analysis and subsequent experiments.

The RAA assay targeting the *16S rRNA* gene of BCC has the ability to detect a minimum template of 10 copies/µL, and the sensitivity is similar to that of RAA detection of other pathogens (Xiao et al., 2021), which was more sensitive than conventional PCR, qPCR and LAMP assays of BCC (Daddy Gaoh et al., 2021). Compared with conventional PCR ($45/sample, >2 h detection speed) (Pliakos et al., 2018), RAA has a lower cost ($5/sample), faster detection speed (10 min) and simpler reaction conditions, not requiring complex instruments. For samples with 10 copies/µL of BCC, a positive result can be obtained within 10 min under a constant temperature of 39°C. The RAA assay in this study did not cross-react with eight other species of pathogens tested, including one species of non-BCC *Burkholderia* and seven clinically-common species. Of note, we employed four species of the BCC and one non-BCC *Burkholderia* species (*B. gladioli*) to verify the specificity of the method, and the results showed that this method can effectively distinguish between BCC and non-BCC species.

To evaluate the clinical applicability of this method, we further collected 269 clinical samples for BCC detection. The assay had a sensitivity of 100% and a specificity of 98.5%, which was superior to other reported assays (Daddy Gaoh et al., 2021). In particular, the four samples that tested negative for conventional PCR but positive for RAA also exhibited low peak values for the RAA assay. Moreover, previous studies have shown a low rate of infection with BCC (Baidya et al., 2021), but the results of the RAA assay in this study suggest a higher prevalence. BCC screening in specific populations may be strengthened through the application of the RAA assay in the future.

We carried out a case study of a child with *B. vietnamiensis* infection to test the applicability of RAA for clinical detection. The patient underwent recurrent infection as a result of congenital immunodeficiency, chronic granulomatous disease and the heavy use of antibiotics. The RAA assay in this study was able to detect *B. multivorans*, *B. vietnamiensis* and other common clinical BCC members (Rhodes and Schweizer, 2016). Thus, if patients with a similar course of disease were observed in clinic in the future, our RAA assay could be applied to quickly detect BCC infection. It is worth noting that the treatment of BCC infection relies on ceftazidime and other broad-spectrum cephalosporins (Rhodes and Schweizer, 2016). Although imipenem (a broad-spectrum β-lactam antibiotic) can be effective with clinical recovery occurring at a later stage, doctors often employ a large number of antibiotics to target a range of pathogens when the causative pathogen is unknown at an early stage. In the future, the aim would be to achieve early and rapid pathogen detection through RAA, enabling early targeted treatment and thereby avoiding the overuse of antibiotics.

Taken together, we established an RAA assay for BCC detection, which has high specificity and sensitivity. RAA has the advantages of simple reaction conditions, a short reaction time and low cost. The applicability of the assay was also verified in clinic. However, future studies are needed to analyze a greater number of clinical samples to further verify the effectiveness of the assay in the early detection of BCC infection.

## Data availability statement

The datasets presented in this study can be found in online repositories. The names of the repository/repositories and accession number(s) can be found in the article/**Supplementary Material**.

## Ethics statement

The studies involving human participants were reviewed and approved by This study was performed in compliance with the Helsinki Declaration (Ethical Principles for Medical Research Involving Human Subjects) and was approved by the research board of the Ethics Committee of the Capital Institute of Pediatrics, Beijing, China (SHERLLM2022004). All specimens used in this study are part of routine patient management without any additional collection, and all patient data were anonymously reported. Written informed consent to participate in this study was provided by the participants’ legal guardian/next of kin.

## Author contributions

JY and LC designed the study and revised the manuscript. HF, LG, ZT, JH, BD, GX, YF, HZ, JC, CY, JF, ZF, TF, ZX, RZ, XC, SD, YZ and QZ performed the experiments. JH and YF collected the clinical samples. HF and LG analyzed the results. HF and LG wrote the manuscript. All authors contributed to the article and approved the submitted version.

## Funding

This work was financially supported by grants from the National Natural Science Foundation (82002191, 82130065 and 32170201), Public service development and reform pilot project of Beijing Medical Research Institute (BMR2019-11) and Feng Foundation (FFBR 202103).

## Conflict of interest

The authors declare that the research was conducted in the absence of any commercial or financial relationships that could be construed as a potential conflict of interest.

## Publisher’s note

All claims expressed in this article are solely those of the authors and do not necessarily represent those of their affiliated organizations, or those of the publisher, the editors and the reviewers. Any product that may be evaluated in this article, or claim that may be made by its manufacturer, is not guaranteed or endorsed by the publisher.

## Supplementary material

Supplementary Figure 1 Four sets of primers and probes used for RAA assay. The DNA template used in this assay was extracted from recombinant plasmid, and the negative control was nucleic acid-free water.
