# Supplementary figures and images for: Rapid detection of Burkholderia cepacia complex carrying the 16S rRNA gene in clinical specimens by recombinase-aided amplification
Source: Front Cell Infect Microbiol. 2022 Sep 5;12:984140. doi: 10.3389/fcimb.2022.984140 (PMC9483118; doi:10.3389/fcimb.2022.984140)

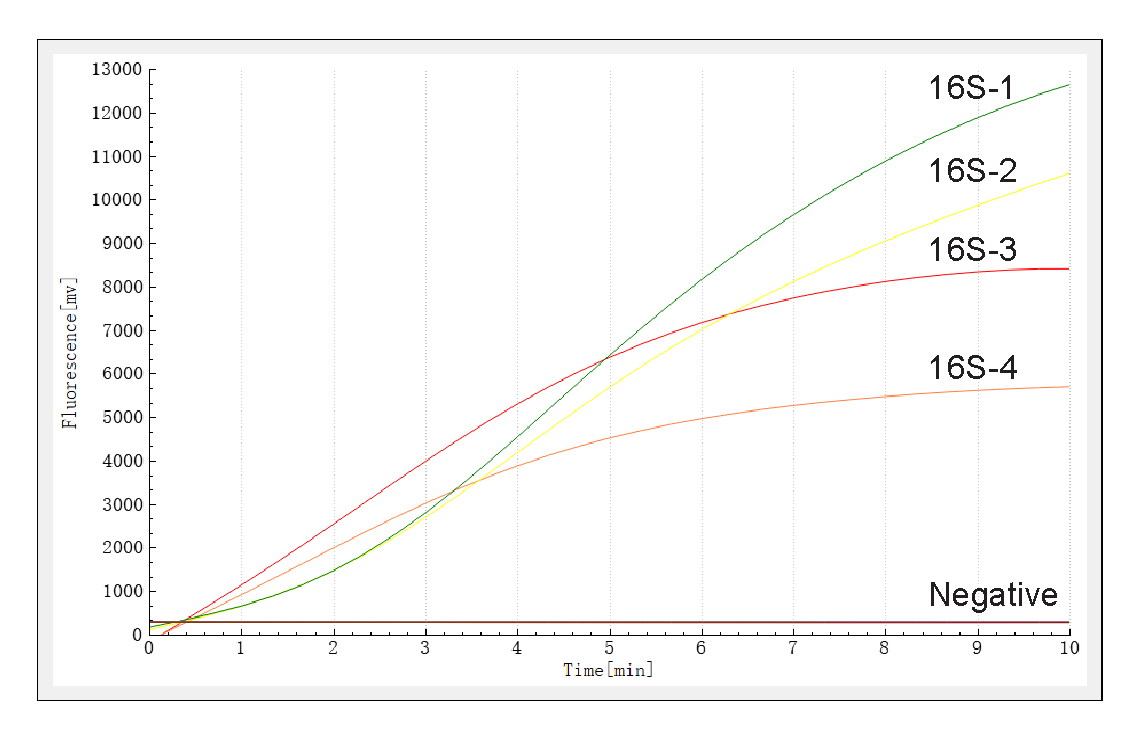

Supplement: Supplementary Figure 1 — Four sets of primers and probes used for RAA assay. The DNA template used in this assay was extracted from recombinant plasmid, and the negative control was nucleic acid-free water. [file Image_1.tif]
